# Blood and skin-derived Sezary cells: differences in proliferation-index, activation of PI3K/AKT/mTORC1 pathway and its prognostic relevance

**DOI:** 10.1038/s41375-018-0305-8

**Published:** 2018-12-05

**Authors:** Cristina Cristofoletti, Antonella Bresin, Mario Picozza, Maria Cristina Picchio, Francesca Monzo, Mauro Helmer Citterich, Francesca Passarelli, Alessandra Frezzolini, Enrico Scala, Alessandro Monopoli, Maria Cantonetti, Roberto Benucci, Stefania D’Atri, Elisabetta Caprini, Giandomenico Russo, Maria Grazia Narducci

**Affiliations:** 1 Istituto Dermopatico dell’Immacolata, IDI-IRCCS, 00167 Rome, Italy; 2 Laboratory of Neuroimmunology, Fondazione Santa Lucia, 00143 Rome, Italy; 3 Department of Hematology, University of Tor Vergata, 00133 Rome, Italy

**Keywords:** Cancer microenvironment, Chemokines, Cancer genomics, T-cell lymphoma

## Abstract

Sézary syndrome (SS) is a rare and aggressive variant of Cutaneous T-Cell Lymphoma characterized by neoplastic distribution mainly involving blood, skin, and lymph-node. Although a role of the skin microenvironment in SS pathogenesis has long been hypothesized, its function in vivo is poorly characterized. To deepen this aspect, here we compared skin to blood-derived SS cells concurrently obtained from SS patients highlighting a greater proliferation-index and a PI3K/AKT/mTORC1 pathway activation level, particularly of mTOR protein, in skin-derived-SS cells. We proved that SDF-1 and CCL21 chemokines, both overexpressed in SS tissues, induce mTORC1 signaling activation, cell proliferation and Ki67 up-regulation in a SS-derived cell line and primary-SS cells. In a cohort of 43 SS cases, we observed recurrent copy number variations (CNV) of members belonging to this cascade, namely: loss of LKB1 (48%), PTEN (39%) and PDCD4 (35%) and gains of P70S6K (30%). These alterations represent druggable targets unraveling new therapeutic treatments as metformin here evaluated in vitro. Moreover, CNV of PTEN, PDCD4, and P70S6K, evaluated individually or in combination, are associated with reduced survival of SS patients. These data shed light on effects in vivo of skin-SS cells interaction underlying the prognostic and therapeutic relevance of mTORC1 pathway in SS.

## Introduction

Sézary syndrome (SS) is a rare aggressive leukemic variant of cutaneous T-cell lymphomas (CTCLs) in which malignant T cells accumulate in the skin, lymph nodes and blood, typically resulting in a shortened life expectancy with a median of survival of 63 months [1, 2].

SS cells express CD45R0 + CCR7 + CD27 + CD62L+ accordingly with a central memory (CM) T cells phenotype representing mature long-lived lymphocytes with a high proliferative and migratory potential [3]. SS cells carry recurrent chromosomal alterations as loss of 17p, 10q, 19p and gains of 17q, 8/8q [4–6] and deep sequencing studies have identified mutations in genes involved in epigenetic, DNA repair, cell cycle, apoptotic and TCR-signaling mechanisms [7–12]. Despite these findings, no specific therapy is available yet to treat SS [13].

SS cells grow poorly in vitro also in presence of multiple cytokines, growth factors, macrophages, dendritic and mast cells indicating that nutrients and signals released by tumor microenvironment are essential to support their proliferation and survival [14–18]. We previously demonstrated that the PTEN, that antagonizes the PI3K/AKT signaling [19], is commonly downregulated in SS [20] and that AKT is mainly activated in skin tumor cells with respect to blood [20]. These data underline how different environments, as skin and blood, may affect SS cells in response to stimulatory or co-stimulatory signals [20].

Here, we compared skin to blood-derived SS cells concurrently obtained from SS patients to investigate the effect of the microenvironment on SS cells in vivo. This approach allowed us to identify the PI3K/AKT/mTORC1 activation in skin-resident SS cells, a pathway already found altered in CTCL by others [21, 22], that we also analyzed at the genomic and biochemical level in SS cell lines and primary tumor cells.

## Materials and methods

### Patients and CTCL cell lines

This study was conducted in accordance with the Declaration of Helsinki and approved by theEthical Committee of the Istituto Dermopatico dell’Immacolata (ID n. 4/CE/2015). Diagnosis of SS was based on described criteria [1]. Matched SS cell derived from blood and skin were concurrently obtained from SS patients and analyzed in parallel. SS cell isolation from blood was performed as previously described [5]. For samples with a TCR-Vβ+ clonality ≥ 90%, CD4+ neoplastic cells were not purified, otherwise we selected them using the CD4+ untouched separation protocol (Miltenyi Biotech, Germany). In all experiments performed in this study, the primary tumor cells were indicated as SS cells. Isolation of SS cells from fresh skin punches of SS patients was performed by overnight incubation at 37 °C in RPMI 1640 supplemented with 10% fetal bovine serum (FBS) (Sigma-Aldrich St. Louis, MO, USA) and 1 mg/ml Collagenase type IV (Worthington, Lakewood, NJ). Skin-resident SS cells were isolated from fresh-frozen OCT-embedded skin biopsies using a laser micro-dissector (PALM Microlaser System, Bernried, Germany). All biopsies were selected from the files of IDI Pathology and specimens were classified according to the EORTC classification [1].

Clinical characteristics of SS patients used in matched analyses, in vitro signaling, cell proliferation assay and chemotaxis are shown in Supplementary Table S1 and S2.

Hut78 (TIB161), H9 (HTB 176) and HH (CRL2105) cell lines established from peripheral blood of CTCL patients were obtained from American Type Culture Collection (ATCC).

### Immunohistochemistry (IHC)

IHC analyses for CD4 (1:20; Monosan,Uden, The Netherlands) and Ki67 (1:100, Dakocytomation, Glostrup, Denmark), were performed as described; [23] detection of phosphorylated (p) P70S6K(Thr389)(1:20.000, Abcam, Cambridge, UK) was done using Immpress HRP-Polymer detection kit (Vector Laboratories, Burlingame, CA) on cytospins of primary SS and H9 cells suspension fixed with 4% of Paraformaldehyde (Sigma-Aldrich) and paired paraffin- skin-biopsies. Double staining was performed as described in Supplementary Information

## Results

### Skin-derived SS cells show a higher proliferation-index than matched circulating SS cells

**Fig. 1:**
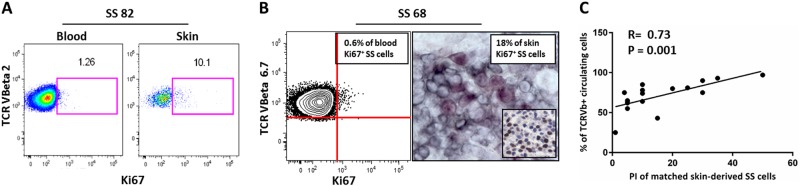
Skin-derived SS cells show a higher proliferation-index (PI) compared to matched circulating SS cells, a data that correlates with tumor burden. **a** Fresh blood and skin-derived cells concurrently obtained from patient SS82 were analyzed by FACS and the percentage of Ki67+ SS cells (i.e., PI) was calculated within the neoplastic clone identified by CD3, CD4 and CCR7 positivity and co-expression of TCR-Vβ2 chain rearrangement; **b** In SS68 patient, PI was measured by FACS within the TCR-Vβ 6.7 clone and in matched OCT skin biopsy by double IHC where KI67+ SS cells were recognized by a co-staining with specific anti-TCR-Vβ6.7 (black) and anti- Ki67 (purple) antibodies; in the insert, IHC for Ki67 performed on matched paraffin-skin biopsy in which neoplastic cells were recognized for morphologic features by hematoxylin counter-staining. In IHC experiments, Ki67+ SS cells were calculated by a pathologist, counting at least 400 tumor cells in high-power fields with a 40x object lens; **c** A positive correlation between matched skin-PI values and tumor burden expressed as expansion of circulating clonal TCR-Vβ+ cells was also observed (*n* = 17, *R* = 0.73, *P* = 0.001 for Spearman test)

We analyzed by IHC the expression of the proliferation marker Ki67 on 17 SS skin biopsies. The percentage of Ki67+ neoplastic cells (the Proliferation Index, PI) varied from 5 to 50% (mean 16.23 ± 13.38). We also measured the PI by FACS on 11 blood-derived SS samples and it ranged from 0.64 to 5% (mean 1.63% ± 1.29). (Table S1). These results indicated that the PI values were clearly different between skin and blood (*P* < 0.001). However, since this counting was evaluated on SS cells deriving from two different compartments by two different techniques, we compared the PI of matched skin and blood samples concurrently obtained from eight SS patients. In these cases, the percentage of Ki67 + SS cells was calculated, using a gating strategy, within the neoplastic clone recognized by the co-expression of CD3, CD4, CCR7 and the specific rearrangement of TCR-Vβ chain by FACS (three samples) as showed in Figure S1 and by double IHC (five samples) (two representative cases are shown in Fig. 1a, b). In these matched cases we confirmed that skin-derived SS cells exhibited a higher PI respect to blood-derived SS cells (*n* = 8, 11 ± 15% versus 1.9 ± 1.4%; fold change (FC) = 5.8; *P* = 0.01). To note that PI values obtained by IHC for  Ki67 were comparable with those obtained by dual IHC (see 8th and 9th columns of the Table S1). We then correlated the PI values of the 17 SS paraffin-skin biopsies with matched clinical data available as CD4/CD8 ratio, eosinophil count, percentage of circulating clonal TCR-Vβ+ cells and survival. Results obtained revealed a significant direct relationship between PI of skin-derived SS cells and the tumor burden expressed as percentage of circulating clonal Vβ+ cells concurrently measured in these individuals (*n* = 17, *R* = 0.73, *P* = 0.001) (Fig. 1c and Table S1).

### Skin-derived SS cells show a higher level of mTOR activation than matched circulating SS cells

**Fig. 2:**
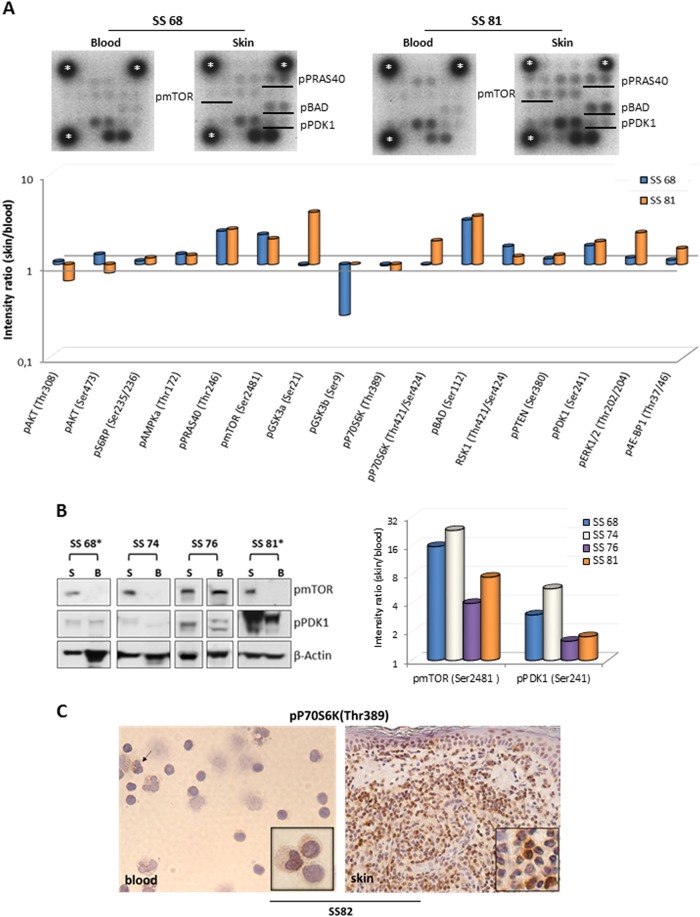
Comparison of phosphorylation levels of members of PI3K/AKT pathway between matched circulating and skin-resident SS cells. **a** upper panel, a phospho-proteins kinase array was probed with protein lysates of blood and skin-derived SS cells concurrently obtained from 2 SS patients. The dots corresponding to p-mTOR, p-PRAS40, p-BAD and p-PDK1 were indicated with black lines. Housekeeping protein is represented by *. **a** lower panel, graph represents the densitometric values of dots corresponding to each protein normalized to housekeeping and expressed as fold change (FC) with respect to matched blood SS cells. **b** left panel, WB validation for indicated proteins was accomplished in matched skin (**S**) and blood (**B**) SS cells of four SS patients. Because of the timing of patient recruitment, the gels were run at separate times and the lanes were composed for the  figure. **b** right panel, graph represents the densitometric values of normalized phosphorylated proteins expressed as FC with respect to matched blood SS cells. **c** IHC for pP70S6K shows a weak positivity in 4% of circulating SS cells and an intense positivity in about 70% of skin-resident SS cells concurrently obtained from patient SS 82. Inserts show SS cells at higher magnification. A weak positivity is also observed in neutrophil cells (arrow)

Next, we analyzed in detail the pathway of PI3K/AKT/mTOR that regulates proliferation and survival in matched skin and blood SS cells. For this purpose, the protein of blood and skin purified SS cells of SS68 and SS81 individuals were used in a pairwise-comparison of phospho levels of 16 members belonging to PI3K/AKT/mTOR pathway [24] using a kinase array (Fig. 2a). The results, expressed as FC values calculated using the matched circulating SS cells as reference, indicated an enhanced activation of many components of this cascade in skin-derived cells. Higher skin FC values (from 1.61 to 3.38) were observed in both patients for PRAS40(Thr246), an inhibitory subunit of mTORC1, mTOR(Ser2481), that phosphorylated in this position may represent both mTORC1/2 complexes [25, 26] BAD(Ser112) that, in this status, abrogates its pro-apoptotic activity and PDK1(Ser241) which is a 3-phosphoinositide dependent kinase traditionally linked to AKT activation [24] (Fig. 2a).

We found, only in patient SS81, a hyper-phosphorylation of the multifunctional kinase GSK3A(Ser21) (FC = 3.73), of p70S6K(Thr421/Ser424) which is a direct downstream member of mTORC1 (FC = 1.81) and of ERK1/2, a central component of RAS/MAP kinase signaling involved in motility and proliferation (FC = 2.23). With the exceptions of GSK3B(Ser9) showing higher activation in circulating cells with a FC of 0.28 in SS68, all other members of the cascade displayed comparable phosphorylation levels between blood and skin-derived SS cells (Fig. 2a).

Among the more skin-activated members, we validated mTOR and PDK1 on four patients (Table S1). Results obtained confirmed a higher phospho levels in skin of mTOR(Ser2481), with FC values ranging from 3.9 to 22.9, and PDK1(Ser241) with FC ranging from 1.6 to 5.6 in all samples (Fig. 2b left and right  panel). Validation of pERK confirmed its skin hyper activation in all three patients analyzed with FC values ranging from 1.2 to 12.8 (Fig. S3). Additionally, we evaluated the activation of mTORC1 signaling by IHC detection of its downstream member, pP70S6K(Thr389), on additional three paired skin-blood samples (Table S1) and H9 cells used as positive control (Fig. S4). Compared to above kinase data, pP70S6K(Thr 389) appeared more expressed in skin respect to matched blood SS cells in all three cases studied possibly reflecting a clinical heterogeneity and/or different therapeutic treatments among patients analyzed and/or the different techniques used to perform these experiments. One out of three cases analyzed is showed in Fig. 2c.

### SDF-1 and CCL21 chemokines activate mTORC1 pathway, promote tumor proliferation and up-regulate the Ki67 expression in H9 cell line and primary SS cells

**Fig. 3:**
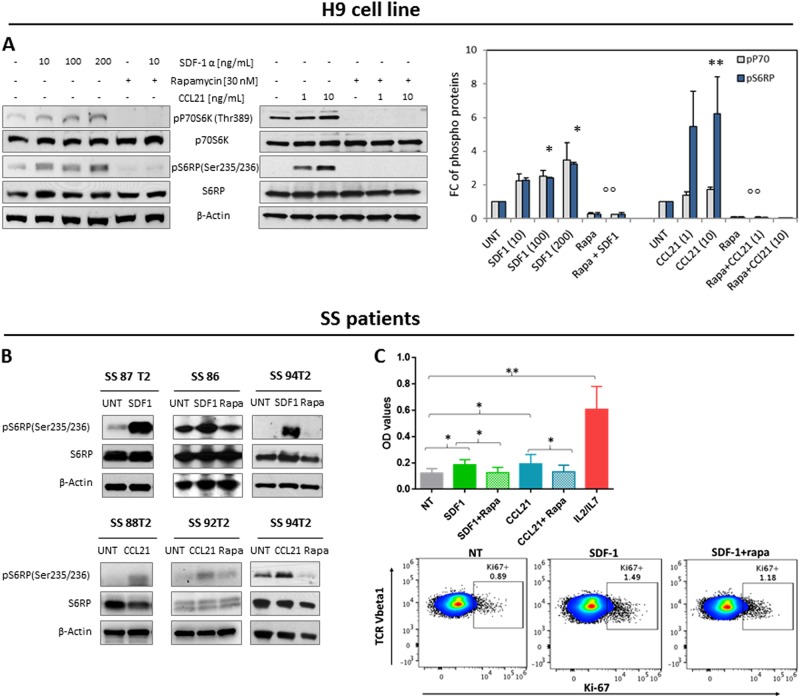
SDF-1 and CCL21 chemokines activate mTORC1 pathway, promote tumor proliferation and up-regulate the Ki67 expression in H9 cell line and primary SS cells. **a** left panel, serum-starved H9 cells were left untreated (UNT) or pre-incubated with rapamycin for 2 h and then stimulated or not with SDF-1 or CCL21 for 30 min at indicated concentrations. WB was performed for the specified proteins. **a** Right panel, densitometric data normalized to β-actin were expressed as mean ± SEM of three independent experiments. Graph shows phospho protein levels expressed as FC respect to UNT samples *P ≤ 0.02, **P = 0.05; °°*P* ≤ 0.0001; **b** upper panel, SS cells from patients cultured in complete medium at high density, left UNT or pre-incubated with 30 nM of rapamycin for 2 h and then stimulated or not for 30 min with 100 ng/ml of SDF-1 or **b** lower panel,with 10 ng/ml of CCL21. WB was performed for the specified proteins. Because of the timing of patient recruitment, the gels were run at separate times and the lanes were composed for the figure. **c** upper panel, SS cells were cultured in vitro at high density for 5 days in complete medium used alone (NT) or supplemented with 300 ng/ml of SDF-1 or CCL21 in presence or absence of rapamycin used at 30 nM or IL2 at 30U/ml plus IL7 at 10 ng/ml. Cell proliferation was determined by MTT assay and data are expressed as means of OD values ± SEM. Statistics were calculated as paired t-test. *P = 0.05; **P = 0.01; **c** lower panel, cell-activation induced by 300 ng/ml of SDF-1 was evaluated in SS cells obtained from SS94 patient, by Ki67 detection using FACS within neoplastic clone recognized by CD3,CD4, CCR7 and TCR-Vβ1 chain rearrangement

mTOR is a central environmental sensor that integrates survival/proliferation and metabolism signals. It represents the catalytic subunit of TORC1 and TORC2, two distinct complexes linked by a negative feedback loop, able to regulate different functions and downstream targets [27].The above described results indicated that both complexes are activated in skin-derived SS cells; however, the finding that TORC1 pathway is triggered in cancer cells by SDF-1 and CCL21 [28–30], two chemokines highly expressed in SS tissues and able to chemoactract SS cells in vitro [23, 37] prompted us to investigate in detail this mechanism. To mimic skin conditions, we stimulated the H9 cells and primary SS cells with SDF-1 and CCL21 to analyze the activation level of mTORC1 downstream members. A significant increase of the level of phosphorylation of P70S6K (pP70S6K) and S6RP (pS6RP) was observed after 30 min of H9 stimulation with both chemokines compared to untreated cells. Both phosphorylations were significantly inhibited by a 2 h pretreatments with rapamycin, the specific inhibitor of mTORC1 [31] (Fig. 3a). As P70S6K can directly phosphorylate PDCD4 promoting its degradation and releasing the inhibitory effect on the initial step of protein translation [32], we next evaluated the phosphorylated and non-phosphorylated form of this protein in H9 cells exposed to SDF-1 for different times. After 30 min we observed an increase of pPDCD4 level that was inhibited by 2 hours (h) of rapamycin pretreatment whereas an increase of PDCD4 level, able to block protein synthesis [32], was observed after a longer rapamycin exposure peaking after 24 h (Fig. S5). Then, to verify that mTORC1 activation occurs also in primary malignant lymphocytes we analyzed freshly circulating SS cells from five patients (Table S2). Also in this system we observed an increase of pS6RP level in response of 100 ng/ml of SDF-1 and 10 ng/ml of CCL21 that was inhibited by rapamycin (Fig. 3b). Lastly, we wondered if these chemokines also promoted SS cells proliferation. Purified SS cells from 5 patients (Table S2), cultured at high density in complete medium with or without SDF-1 or CCL21, demonstrated that both chemokines significantly enhanced proliferation, although to lesser extent than T-cell growth factors like IL2/IL7; we assessed that rapamycin inhibited this chemokine-induced effect (upper panel, Fig. 3c). In two cases (Table S2), we also evaluated the cell-activation status induced by SDF-1 demonstrating that it up-regulated the Ki67 expression while rapamycin inhibited it (one representative case is shown in lower panel of Fig. 3c). Even if with less efficacy, CCL21 showed similar results (Fig. S6).

### SS patients show frequent genome copy number variations (CNVs) of members of the PI3K/AKT/mTORC1 pathway

**Fig. 4:**
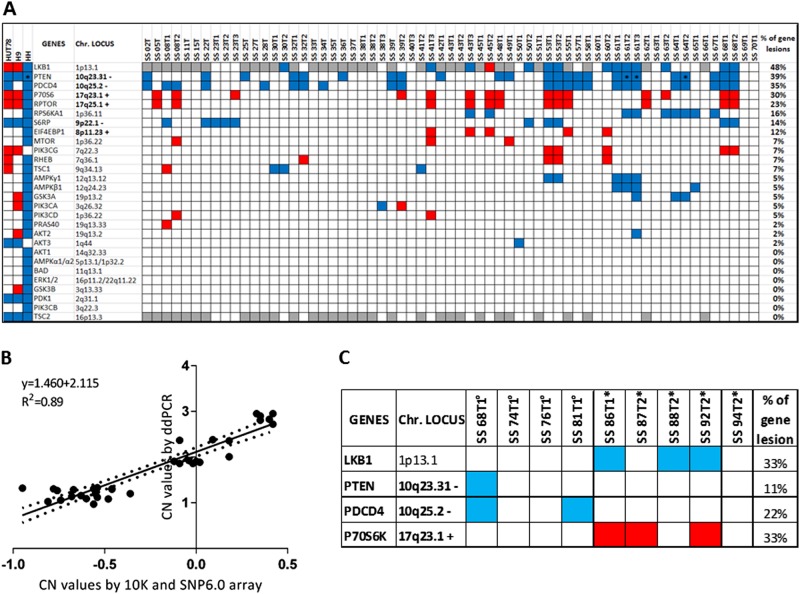
Frequency of CN changes of members belonging to PI3K/AKT/mTOR pathway observed in a cohort of 43 SS patients. **a** CN data were obtained using Affymetrix 10 K and SNP6.0 platforms. Monoallelelic gains and losses are represented in red and blue, respectively. Bi-allelic loss is indicated as*. Genes not covered by 10 K array are represented in gray. Percentages reflect the lesion frequency observed for each gene. **b** CN values for PDCD4, LKB1 and P70S6K genes obtained by ddPCR versus 10 K/SNP6.0 arrays on a total of 30 samples plus diploid reference gDNA (Affymetrix) used as controls. Graph is based on a linear regression model. Solid line indicates the fitting curve. Dotted line represents 95% confidence limits. *P* = < 0.0001. **c** CN values for LKB1, PTEN, PDCD4 and P70S6K obtained by ddPCR on patients analyzed for kinase array validation (°) and mTORC1 in vitro signaling (*). Losses are represented in blue, gains in red

Since we previously demonstrated that PTEN is deleted in 36% of SS individuals but no mutation was detected within its coding region [20], we wondered if and how often additional genetic mechanisms potentially leading to PI3K/AKT/mTOR pathway dysregulation occurred in SS. With this aim we investigated the CN status of the main members of this cascade using the Affymetrix arrays data of circulating tumor cells derived from 63 SS samples (43 patients plus 20 F-UP) and 3 CTCL cell lines [20]. We observed that many components of PI3K/AKT/mTOR pathway showed frequent deletions and amplifications that, with few exceptions observed for PTEN, appeared as monoallelic defects (Fig. 4a). We detected recurrent deletions involving TS genes representing upstream negative regulators of mTOR signaling: we found loss of LKB1 in 9 of 21 cases (48%) as measured by the higher genome resolution SNP6.0 platform and we confirmed PTEN deletion in 17 of 43 cases (39%) in agreement with our previous study [20]. Among the downstream members of mTORC1 pathway we found recurrent deletions of the TS PDCD4 in 15 of 43 cases (35%), gains of the proto-oncogene P70S6K in 13 of 43 (30%) and of RAPTOR in 10 of 43 cases (23%) and deletions of P90S6K in 7 out 43 cases (16%) (Fig. 4a).

Moreover, we found CN variations (CNV) of two other downstream effector members of mTORC1: loss of S6RP in 6 of 43 (14%) and the gain of EIF4EBP1 (alias 4EBP1) in 5 of 43 cases (12%). Other components of this pathway including mTOR, PIK3CG, RHEB, TSC1, AMPKG1, GSK3a, PI3KCA, PI3KCD, and PRAS40 showed a CNV at lower frequencies ranging from 2.3 to 7%, whereas the remaining pathway components did not show any CN changes. CN abnormalities of multiple members of this signaling co-occurred also in H9, Hut78 and HH cell lines as shown in the left columns of Fig. 4a.

We validated CN values found for PDCD4, LKB1and P70S6K in 10 patientsby ddPCR. We obtained values ranging from 0.97 to 1.33 for CN loss (PDCD4 and LKB1), from 2.72 to 2.95 for CN gain (P70S6K) and from 1.85 to 2.38 for WT conditions (Table S3**)**. Thus, ddPCR values were in agreement with those obtained with 10 K and SNP6.0 arrays as demonstrated by Pearson’s correlation (*R* = 0.94, *P* < 0.0001) and the linear regression (*P* = 0.0001) (Fig. 4b).

Based on these results, we demonstrated by ddPCR that CN changes of PTEN, PDCD4, LKB1 and P70S6K occur also in SS patients used for phospho-array validation (Fig. 2c) and TORC1 activation signaling in vitro (Fig. 3b) as showed in Fig. 4c. Moreover, we confirmed CN changes of LKB1, PDCD4 and P70S6K by ddPCR also in the skin-resident SS cells of three cases for whom we had genomic DNA: SS30T2, SS60T1 and SS81T1. The same CN status of these genes detected in circulating and skin-derived SS cells of these patients (Table S3**)** indicated that these two cellular subsets show a homogeneous genomic phenotype.

### Multiple CNV of members of the PI3K/AKT/mTOR pathway define survival classes in SS

**Fig. 5:**
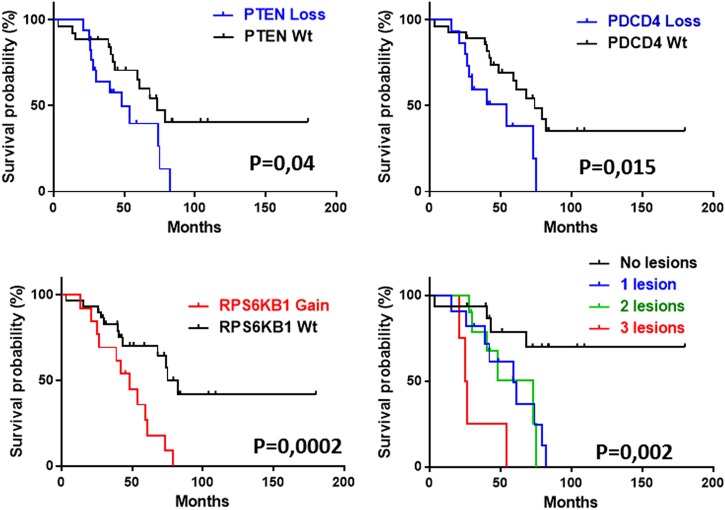
CN variations of PTEN, PDCD4 and P70S6K correlate with the outcome and define survival classes in SS. SS patients were identified by CNV of PTEN (*P* = 0.04), PDCD4 (*P* = 0.015) and P70S6K (*P* = 0.0002) and analyzed by Kaplan Meier (KM) analysis. KM performed on four clusters of patients identified from the total number of lesions observed for these genes (from 0 to 3) showed a progressive shorter survival with increasing number of lesions (*P* = 0.002). Statistic was calculated by Log-rank test

Patients with CN loss of PTEN (*n* = 17), PDCD4 (*n* = 15) and CN gain of P70S6K (*n* = 13) revealed an unfavorable outcome compared to individuals with the corresponding WT status of these loci showing a median of overall survival (OS) of 48.4 months vs 73.1 months. (*P* = 0.04), 54 vs 74 months (*P* = 0.015) and 48.4 vs 82 months (*P* = 0.002), respectively (Fig. 5). No significant difference in survival was seen for LKB1 deletions (data not shown) probably because of the smaller sample size analyzed by SNP6 array (*n* = 23). Kaplan Maier (KM) analysis revealed also a significant difference in survival when patients were clustered in four distinct groups associated to: (a) no lesions found for PTEN, PDCD4 and P70S6K (*n* = 16); (b) a lesion affecting one gene of three (*n* = 13); (c) lesions involving two genes of three (*n* = 10); (d) lesions including all three genes (*n* = 4).

The resulting KM analyses demonstrated that SS patients without lesions in three genes were long survivors, those displaying one or two lesions showed a progressive shorter outcome with a median OS of 73 and 59 months, respectively, whereas patients with three lesions were associated with the worst clinical outcome with a median OS of 25.7 months (*P* = 0.002) (Fig. 5).

### Metformin inhibits mTORC1 signaling and migration of SS cells  induced by SDF-1

**Fig. 6:**
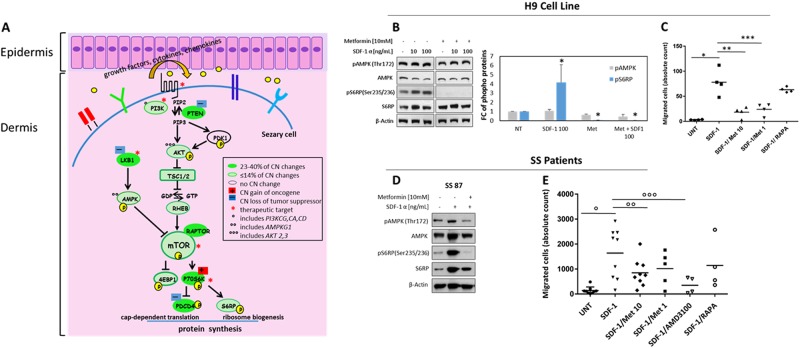
Diagram of skin interaction with Sezary cells and effects of metformin in H9 cell line and primary SS cells. **a** Map of PI3K/AKT/mTORC1 pathway including members CN changes found in 43 SS individuals. Asterisks indicate therapeutic targets; **b** left panel, H9 cells untreated (UNT) or pre-treated for 2 h with metformin at 10 mM and then stimulated or not for 30 min with SDF-1 at 10 and 100 ng/ml were analyzed by WB for indicated proteins as showed by one representative experiment. **b** right panel, densitometric data normalized to β-actin were expressed as mean ± SEM of 3 independent experiments. Graph shows phospho protein levels expressed as FC respect to UNT samples *P = ≤ 0.05**. c** H9 cells UNT or pre-treated for 2 h with metformin at 10 mM or 1 mM or rapamycin at 30 nM were allowed to migrate in response of SDF-1 used at 300 ng/ml. **d** SS cells left UNT or 2 h-pretreated with metformin and then stimulated with SDF-1 at 100 ng/ml were analyzed by WB as above described as showed by one representative experiments; **e** SS UNT or pre-treated for 2 h with 10 mM or 1 mM of metformin or AMD3100 at 15 μM or rapamycin at 30 nM were allowed to migrate in response to SDF-1 at 300 ng/ml. In both systems, migration results are shown as an absolute number of CD4^+^ migrated cells measured by flow cytometry. Each dot represents a sample; bars represent the mean of migration counts. *P = 0.01, ° *P* = 0.0014; **and °° and ***; °°°*P* ≤ 0.05 were calculated by paired *t*-test

Genome alterations found here suggest that the losses of TSs as PTEN, LKB1 and PDCD4 as well as the gain of the oncogene P70S6K might converge toward an increased oncogenic activity of PI3K/AKT/mTORC1 signaling and that many members might represent novel therapeutic targets to treat SS (Fig. 6a) [33]. Among these, we focused on TS LKB1, that represents the most altered gene with a loss in 48% of cases studied. LKB1 is a kinase that activates AMPK that, in turn, inhibits the mTOR signaling [34]. Drugs able to activate AMPK are therefore useful to fight the hyperactive mTORC1 signaling and one of the most commonly used is metformin, a repurposing antidiabetic drug with direct antitumor properties [35]. Beyond glycemic control, metformin inhibits mTORC1 signaling by AMPK activation via LKB1 or by an AMPK-independent mechanism, for example in a ragGTPase-dependent manner [35, 36]. As the recurrent CN loss of LKB1 here described might prejudice the LKB1/AMPK axis function in SS, we wondered if metformin could have an effect on mTORC1 signaling in this neoplasia. With this purpose, we analyzed metformin pretreated H9 cells (*n* = 3) and primary SS cells (*n* = 2) stimulated or not by SDF-1 that we found more abundant in SS skin than CCL21, which is mainly expressed in endothelial cells [37]. In both systems, 2 h-exposure of metformin prevents mTORC1 activation as demonstrated by the inhibition of S6RP phosphorylation by a mechanism that appears to be AMPK-independent (Figs. 6b, d and Fig. S7).

Metformin also inhibits chemotaxis of cancer cells through mechanisms still not well understood [38]. As SS cells express several chemokine receptors including CXCR4 and CCR7 [23, 39] and they migrate in vitro in response to SDF-1 and CCL21 [23, 37], we investigated the effect of metformin on SS cells chemotaxis. Using a transwell assay we observed that metformin at 10 mM and 1 mM, significantly inhibited H9 cell migration towards SDF-1 whereas a minor effect was observed with 30 nM rapamycin pretreatment (Fig. 6c). Similarly, SS cells obtained from patients (Table S2) were significantly inhibited with pretreatments of metformin used at 10 mM (*n* = 9) or 1 mM (*n* = 5) (Fig. 6e). A significant inhibition was also observed with 15 μM of AMD3100, an antagonist of CXCR4 that we used to verify the specificity of CXCR4-SDF-1 binding(*n* = 4) whereas rapamycin at 30 nM interfered less also with chemotaxis of SS cells (*n* = 4) (Fig. 6e). This lower effect suggests that rapamycin, beyond mTOR pathway [30, 40], interferes less with other signaling(s) involved in locomotion respect to metformin. We also proved that metformin inhibits chemotaxis toward CCL21 (Fig. S8).

Lastly, in agreement with the cytostatic effect of metformin, we observed a significant reduction of cell viability in CTCL cell lines but not in low proliferating primary circulating SS cells after metformin treatment, as demonstrated by MTT and FACS analysis (Fig. S9).

## Discussion

Previous studies have described SS cells as quiescent and apoptotic-resistant malignant lymphocytes classifying SS principally as an accumulative disorder [15, 41]. Although earlier studies using tritiated thymidine labeling in vivo generically indicated a higher SS cells proliferation in skin and lymph nodes [42] here we demonstrated, with more accuracy, to what extent the skin-resident SS cells proliferate more respect to those of blood using the Ki67 analysis, a widely recognized marker of cell activation/proliferation in daily pathologic practice [43]. We also observed that skin PI increases with the expansion of tumor burden measured as blood TCR-Vβ clonality indicating that skin and blood compartments are interconnected, thus supporting the concept that activation/proliferation plus apoptosis resistance could determine the SS clinical outcome.

Another key purpose of this study was to identify which members of the PI3K/AKT/mTOR signaling are engaged in vivo in SS. Using a skin-blood comparison approach, we found that skin-derived SS cells mainly show a higher level of mTORC1/mTORC2 activation than corresponding circulating SS cells. In CTCL, the involvement of TORC1 is already demonstrated by studies conducted on rapamycin, a compound that mainly inhibits this complex [31], able to reduce proliferation of CTCL cell lines, primary tumor cells [21] and tumor size in xenograft mice [22]. Recent investigations demonstrated that SDF-1 and CCL21 chemokines through their cognate receptors CXCR4 and CCR7, signals to mTOR pathway in other cancers [28–30]. As SS cells express several chemokine receptors [23, 37, 39] including CXCR4 which is involved in metastatic processes and CCR7 that is responsible for lymph-node homing [44], we demonstrated that both SDF-1 and CCL21 activate mTORC1 also in this malignancy. The importance of this finding is supported by the ability of SS cells to migrate in response to SDF-1 and CCL21/19 which are overexpressed (SDF-1 over CCL21) in SS skin [37]. Since SS cells do not express SDF-1 mRNA, but rather they seem to uptake the SDF-1 released from epithelial, dendritic and endothelial cells of the skin [23], we can speculate that SS cells move toward skin through a chemotactic gradient [23]. This hypothesis is further reinforced by the lack from SS cell surface of CD26 peptidase able to cleave and inactivate SDF-1 [23], by the versican overexpression, a proteoglycan that enhances the SS locomotion toward SDF-1 [45] and by the comparable levels of plasmatic SDF-1 found between HDs and SS individuals (*data not shown*).

These results underline that, beyond migration, these chemokines activate the TORC1signaling, pointing toward processes as protein translation and cellular energy [46]. Moreover, similarly to what demonstrated on healthy CD4+ lymphocytes [47, 48] SDF-1 and CCL21 promote proliferation and up-regulate Ki67 expression in SS cells underlying the more activated phenotype of skin resident respect to blood SS cells recently observed by Roelens et al. [49]. Rapamycin inhibited both processes indicating that both SDF-1 and CCL21 induce activation/proliferation by a mTORC1-dependent mechanism(s) that have yet to be clarified

Using SNP technology we also explore the PI3K/AKT/mTOR pathway at the genomic level. We found multiple CNV of members belonging to this cascade that map within large genomic regions described as recurrently unbalanced in SS as 10q and 17q chromosome (chr) [4–6]. Accordingly with these findings, we observed concurrent CN losses of PTEN and PDCD4, both localized at chr 10q23-25, in 30% of the cases as well as concomitant CN gains of P70S6K and RAPTOR, both mapping at chr 17q23-25 in 23% of the samples analyzed. In addition, it is interestingly to note that CNV of the pathway downstream members represented by RPS6 and 4EBP1 genes, respectively involved in cap-dependent translation and ribosome biogenesis [32, 46], occurred in mutually exclusive manner; thus suggesting that SS patients may cluster respect to these gene alterations leading toward different routes of protein synthesis. CNVs assume a very important role in SS since they appear more common than TS/oncogene mutations with respect to other cancers [50]. Accordingly with this feature, the SNPs [20] and the latest NGS analyses [9, 11] revealed no or very few mutations within PTEN coding region of SS individuals while the CN loss of this gene, either in mono or biallelic defect, is a frequent event ranging from 20 to 36% of cases [10, 12, 20]. Absence or very low frequency mutations detected by NGS in LKB1, P70S6K and PDCD4 genes in CTCL/SS lymphomas [10, 12] furthermore support this hypothesis. The importance of CNV in SS is also reinforced by the significant correlation between CN changes and overall survival here demonstrated, indicating these alterations as novel prognostic markers that potentially reflect the alterations of the 10q23 and 17q23 loci on which they map.

mTORC1 regulates protein synthesis and metabolism, two interconnected routes which appear strongly compromised in SS. In fact, in this study we found CNV of both P70S6K and PDCD4 that might reflect a dysfunction of mTORC1-dependent protein synthesis leading to an increased cell size that might explain the larger SS cell dimensions often observed [1, 51]. Moreover, we detected recurrent loss of PTEN that under normal conditions attenuates the upstream activation of mTORC1 through PIP3 dephosphorylation [52] and loss of LKB1, that normally is able to activate AMPK, a kinase that inhibits mTORC1 in low energy conditions [34, 53]. These genomic defects might result in stoichiometric unbalance ultimate leading to a constitutive TORC1 activation promoting a metabolic shift: from oxidative phosphorylation, mainly observed in quiescent/memory lymphocytes toward aerobic glycolysis that increase the glucose demand (Warburg effect) typically observed in activated lymphocytes [53]. Accordingly, rapamycin inhibits tumor growth decreasing aerobic glycolysis in a mouse model of CTCL [54]. Considering the trafficking ability of SS cells [23, 37, 39] these genomic dysfunctions promoting aerobic glycolysis and protein translation might be functionally useful, in term of energy, during the SS cells recruitment to skin and/or lymph node by SDF-1 and CCL21, both able in turn, to promote mTORC1 activation signaling and proliferation of these cells. In summary, these results indicate that the genomic changes here identified might be insufficient to induce SS cell proliferation and emphasize the role of factors present within the skin like cytokines [55], antigens able to engage T-Cell Receptor [56] and the SDF-1 and CCL21 chemokines here studied.

Members of this signaling with CN changes represent, for SS, both novel (as LKB1, P70S6K) and already known (as PI3K, mTOR) therapeutic targets [33]. Actually, the efficacy of rapalogs and dual PI3K/mTOR inhibitors have been largely proven in vitro in T-cell lymphoma [57, 58] but, despite these results, few clinical trials in CTCLs are ongoing (http://www.tcllfoundation.org/current-clinical-trials. Detection of several genomic alterations in PI3K/AKT/mTORC1 pathway indicates that other specific compounds can be tested as the metformin that we demonstrated to inhibit in vitro mTORC1 activation and chemotactic ability of SS cells. Recognition of these lesions might, moreover, improve patient stratification for clinical trials enrollment of SS patients.

## Electronic supplementary material

Supplemental material
