# Supplementary material for: Blood and skin-derived Sezary cells: differences in proliferation-index, activation of PI3K/AKT/mTORC1 pathway and its prognostic relevance
Source: Leukemia. 2018 Dec 5;33(5):1231–42. doi: 10.1038/s41375-018-0305-8 (PMC6756225; doi:10.1038/s41375-018-0305-8)
Supplement: Supplementary file 1 — Supplemental material [file 41375_2018_305_MOESM1_ESM.docx]

**Blood and skin-derived Sezary cells: differences in proliferation-index, activation status of PI3K/AKT/mTORC1 pathway and its prognostic relevance**

**SUPPLEMENTARY INFORMATION**

**SUPPLEMENTARY MATERIALS AND METHODS**

**Immunohistochemistry (IHC)**

Double staining was performed on OCT skin biopsies with the mouse monoclonal antibody (MoAb) against human TCR-Vβ 2, 5.1, 6.7, 17 (1:50, Beckman Coulter, Indianapolis, USA) and TCR-Vb 13.1 rearrangements (1:30, Santa Cruz Biotechnology, Inc. Texas USA) in combination with the rabbit anti-human Ki67 antibodies (1:1000, NovoCastra Lab Newcastle, UK) using Immpress technology. Images were examined under a Zeiss Axioplan2 microscope (Oberkochen, Germany)

**Flow Cytometry (FACS)**

Peripheral Blood Mononuclear Cells (PBMCs) from SS patients were analyzed for T-cell clonality with a TCR-Vβ panel in combination with CD3, CD4, CCR7 MoAb as previously described in detail^1(pp1906-1907)^. Detection of Ki67 was performed as described^2^. Briefly, Ki67 expression within the neoplastic T-clone(s) was evaluated by PBMCs incubation with Zombie dye (Biolegend) to exclude dead cells from analysis. Cells were then stained with specific FITC or PE conjugated TCR-Vβ (Beckman Coulter) in combination with PerCP-Cy5.5-conjugated anti-CD4 and PE-Cy7-conjugated anti-CCR7 MoAbs (Biolegend). When Skin and Blood cell were stained in parallel, depending on results of preliminary surface staining on freshly obtained material, anti-CD8 FITC (Miltenyi Biotec) and/or anti-CD3 ECD (Beckman Coulter) were also added to the staining reaction and used to maximize electronic purity by further software selection. Finally, cells were permeabilized with FOXP3 Fix/Perm Buffer and then stained with APC-conjugated anti-Ki67 Ab (Biolegend). The gating strategy used to analyzed SS cells is shown in **Fig.S1** We used PBMCs from healthy donors (HD), stimulated or not with 10U/ml of phytohemagglutinin (Sigma Aldrich St. Louis, MO) and Hut78 cells as Ki67+ control (**Fig. S2**). Stained samples were run on a FACSAria cell sorter (BD Biosciences) and data analyzed by FlowJo software (Treestar)

**Western blotting (WB)**

WB analysis was performed as already described ^3^ using antibodies listed in **Table** **S4.** Matched blood and skin-derived cells were analyzed using AKT Array Kit (Cell Signaling Technology, Danvers, MA, USA). Film were scanned on a GS-710 Calibrated Imaging Densitometer and analyzed by means of Quantity One Software Version 4.1.1 (Bio-Rad Laboratories, Hercules, CA) and by Image J open source software.

**PI3K/AKT/mTOR pathway copy number (CN) analysis**

The Affymetrix 10K and SNP6.0 arrays data were used to investigate the CN of members of PI3K/AKT/mTOR pathway as previously illustrated (accession code GSE17595)^3^. Sample dataset was composed of 63 samples derived from 43 patients and three CTCL cell lines. Forty-one SS cases were already included in our previous analysis ^3^. With respect to this study, we have introduced a new enrolled patient plus two additional follow up (F-UP) samples. In order to obtain a higher genome resolution, we have substituted SS40T1/T2 with SS40T3 and SS41T1 with SS41T2/T3 samples analyzed with SNP6.0 platform respect the previous to 10K technology. For a higher consistency of our array samples, we removed SS44T1 and SS46T1 of Table 1 of ^3^ showing a clonal expansion ≤50%. The clinical features of new enrolled cases are shown in **Table S5**.

**Droplet digital PCR**

CN of LKB1, PTEN, PDCD4 and P70S6K genes were determined using the QX200 Droplet Digital PCR (ddPCR) CN Variation Assay (Bio-Rad). Briefly, 4 μl of gDNA (10 ng/μl) was mixed in a 20 μl reaction volume with 2x ddPCR Supermix for Probes (No dUDP), 20x of three target primers/probe in FAM and 20x of two reference primers/probe in HEX (**Table S6**). Droplets were read on the Droplet Reader (BioRad) and analysis was performed using QuantaSoft v1.6.6.0320 (Bio-Rad, Hercules, CA, USA).

**Chemotaxis**

Chemotaxis was performed as previously described in detail ^4 (pp1109)^ Migrated cells were stained with phycoerythrin-conjugated mouse anti-CD4 Ab and counted by FACSCalibur (BD), for 120 seconds at a flow rate of 35 µL/min as described ^4^.

**Cell proliferation, MTT assay and apoptosis**

CD4+ cells purified from blood of five SS patients (Supplementary Table S2) used at 5x10^6^ /ml were cultured in 96-well plate for 5 days in complete medium supplemented or not with 300 ng/ml of SDF-1 or CCL21 or IL-2 at 30U/ml plus IL-7 at 10 ng/ml (all purchased from Miltenyi) in presence or absence of rapamycin (Sigma-Aldrich) used at 30nM. Cell proliferation was evaluated by MTT assay and optical density was measured at 595 nm on a microplate reader (Bio-Rad). Viability of cells treated with metformin were evaluated by MTT assay. Apoptosis was analyzed using the Annexin V/PI assay (eBioscience, MA, USA) by flow cytometry FACSCalibur using CellQuestPro software (BD)

**Statistics**

Statistical analyses were carried out with the software Graphpad PRISM 5 (by GraphPad Software Inc. La Jolla, CA, USA). Differences were evaluated with two tails-paired t-test, Pearson correlation, linear regression test and Kaplan Maier estimator. P≤ 0.05 was considered significant.

**SUPPLEMENTARY TABLES**

**Table S1**

**Table S2**

**Table S3**

**
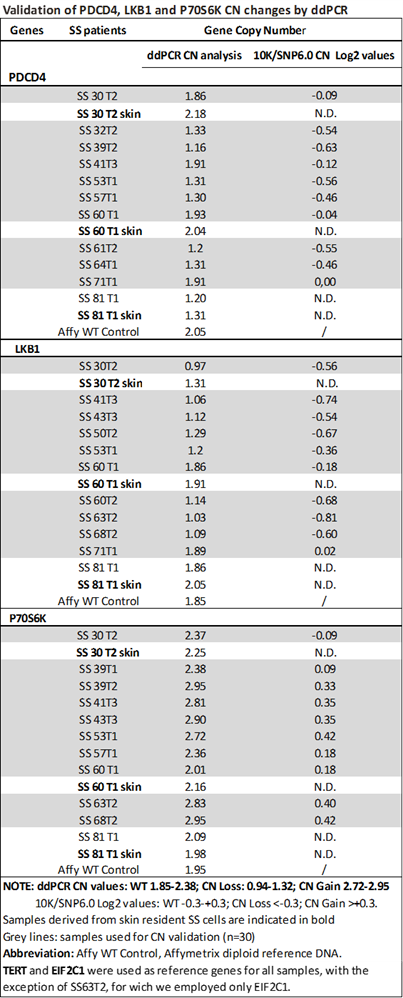
**

**Table S4**

**List of antibodies employed for WB analyses**

| **Primary Antibody** | **Dilution** | **Company** | **Catalogue Number** |
| --- | --- | --- | --- |
| **pmTOR (Ser2448)** | **1:1000** | **Cell Signaling** | **#5536** |
| **pP70S6K (Thr389)** | **1:1000** | **Cell Signaling** | **#9205** |
| **P70S6K** | **1:2000** | **Cell Signaling** | **#2708** |
| **pAMPKα (Thr172)** | **1:2000** | **Cell Signaling** | **#4188** |
| **AMPK** | **1:2000** | **Cell Signaling** | **#2532** |
| **pPDK1 (Ser241)** | **1:2000** | **Cell Signaling** | **#3438** |
| **pPDCD4 (Ser67)** | **1:1000** | **Thermo Fisher** | **PA5-35544** |
| **PDCD4** | **1:1000** | **Santa Cruz** | **sc-130545** |
| **p-p44/42MAPK (T202/Y204)** | **1:1000** | **Cell Signaling** | **#9101** |
| **pS6RP (Ser235/236)** | **1:2000** | **Cell Signaling** | **#2211** |
| **S6RP** | **1:2000** | **Cell Signaling** | **#2317** |
| **β-Actin** | **1:3000** | **Santa Cruz** | **sc-81178** |
| **β-Tubulin** | **1:3000** | **Santa Cruz** | **sc-5274** |
| **Secondary Antibody** | **Dilution** | **Company** | **Catalogue Number** |
| **Anti-mouse IgG** | **1:20000** | **Cell Signaling** | **#7076** |
| **Anti-rabbit IgG** | **1:20000** | **Cell Signaling** | **#7074** |

**Table S5**

| **New SS patients enrolled for SNP6.0 array of this study respect to Cristofoletti and Picchio et al. Blood. 2013** | | | | | | |
| --- | --- | --- | --- | --- | --- | --- |
| **Sample ID** | **Disease** | **Sex/Age** | **TCR-Vß Family** | **TCRVß+ within CD3+CD4+** | **Sample material** | **Array Type** |
| SS 61T3 | SS | M/56 | 23 | 90% | CD4+ sorted cells | SNP 6.0 |
| SS 68T1 | SS | M/69 | 6.7 | 81% | CD4+ sorted cells | SNP 6.0 |
| SS 68T2 | SS | M/70 | 6.7 | 80% | CD4+ sorted cells | SNP 6.0 |

**Table S6**

**SUPPLEMENTARY FIGURES**

**
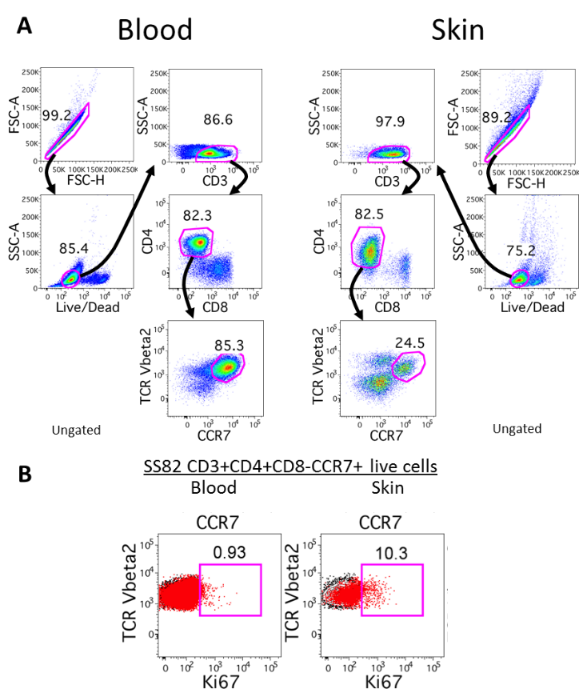
.
Fig. S1 Gating strategy used in a representative SS patient for Ki67 detection in blood and skin-derived-SS cells.** A) Neoplastic cells were selected via sequential gating by the exclusion of doublets and dead cells followed by the positive gating on CD3 and CD4. A further selection for Sezary cells was performed by gating on CCR7/TCR Vβ2 double positive events. B) Ki67 expression was calculated within the neoplastic clone. The number indicate the percentage of KI67+SS cells (Ki67-index) in both compartments.


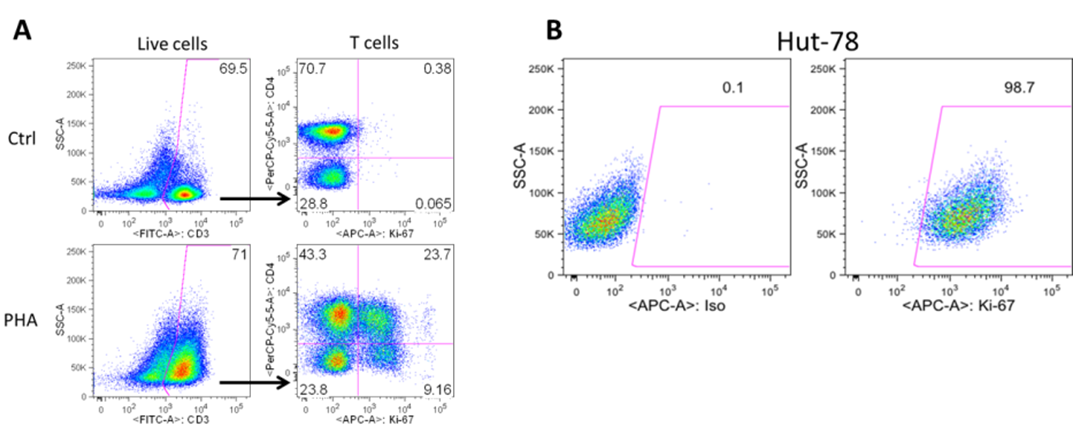


**Fig. S2. Ki67 expression in stimulated in healthy PBMCs and Hut78 cell line.** Ki67 expression was evaluated in PBMCs from healthy donor untreated or stimulated for 3 days with 10U/ml of phytohemagglutinin A) CD3+ cells were gated and visualized in a Ki67 vs CD4 pseudocolor dot plot. B) The assessment of Ki67 was also performed on the continuously growing SS cell line Hut78 to confirm proper intranuclear Ki67 staining of larger cells. Abbreviations: SSC-A, Side Scatter Area; APC, Allophycocyanin; PerCP-Cy5.5, Peridinin Chlorophyll-Cyanine5.5; FITC, Fluorescein isothiocyanate; Iso, Isotype control. Stained samples were run on a FACSAria cell sorter and data analyzed by FlowJo software.

**
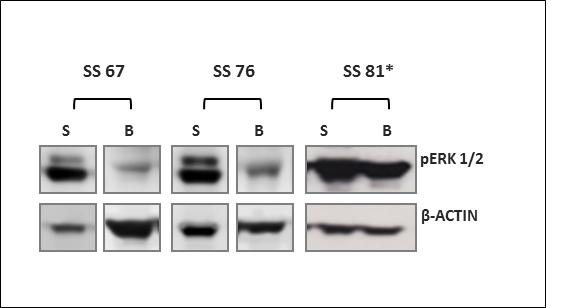
**

**Fig. S3 Validation of pERK1/2 levels by WB**. WB validation was accomplished in matched skin (S) and blood (B) SS cells of 3SS patients. Densitometric values of normalized pERK1/2 expressed as FC respect to paired blood samples are 12.80 for SS67, 4.17 for SS76 and 1.21 for SS81*. *=sample analyzed by kinase array.

**
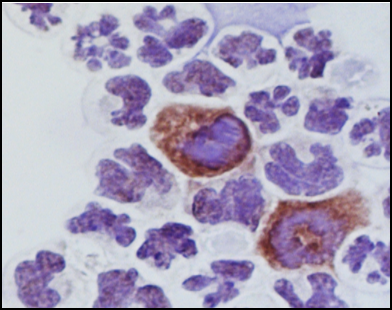
**

**Fig. S4** IHC for pP70S6K showing a strong positivity of H9 cells stimulated with 100 ng/ml of SDF-1 for 30 minutes.

**
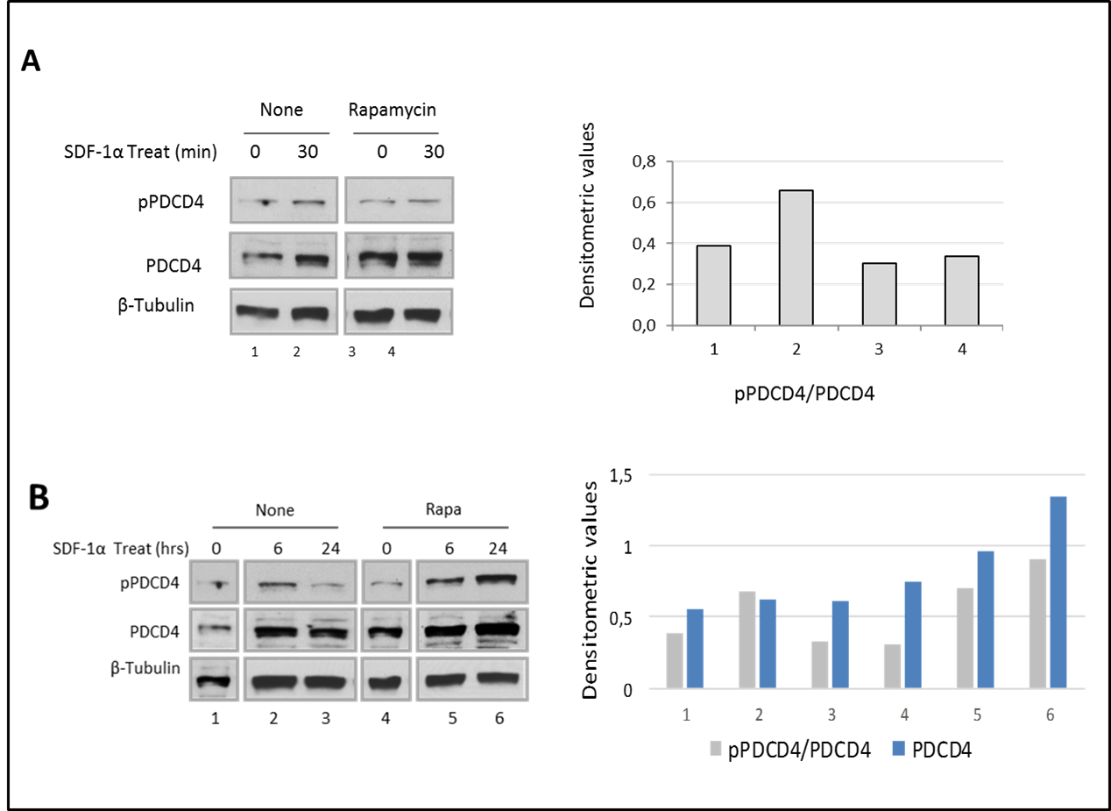
**

**Fig. S5 Effects of SDF-1 on pPDCD4 and PDCD4 level.**  A)Effects of SDF-1 stimulation used at 100 ng/ml in H9 cells left untreated (UNT) or pretreated for 2 hours with rapamycin on A) pPDCD4 level and B) PDCD4 level in H9 cells UNT or pretreated for indicated times with rapamycin. Graphs represent the densitometric values normalized to β-tubulin.

**
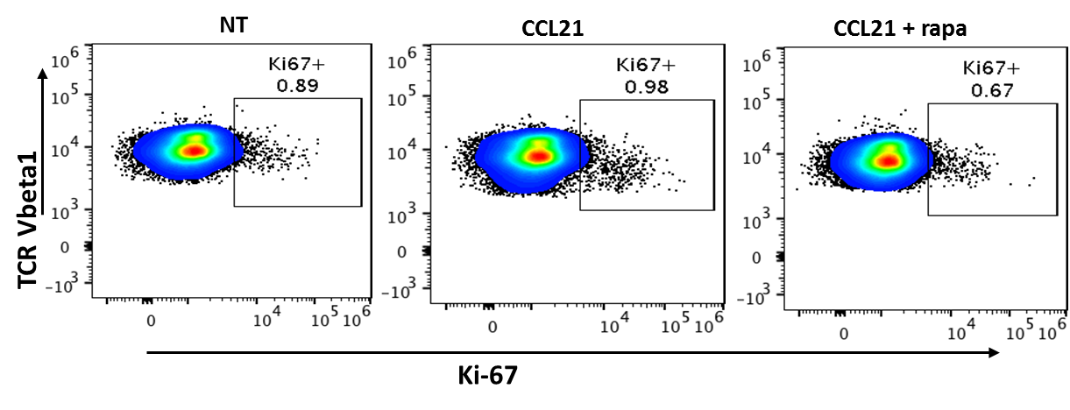
**

**Fig. S6 CCL21 up-regulate Ki67 expression in SS cells.** SS cells obtained from SS94 patient were cultured in vitro at high density for 5 days in complete medium used alone (NT) or supplemented with 300 ng/ml of CCL21 in presence or absence of rapamycin used at 30nM. Cell-activation was assessed measuring the Ki67 expression by FACS within neoplastic clone recognized by CD3,CD4, CCR7 and TCR-Vβ1 chain rearrangement positivity as described in Fig. S1

**
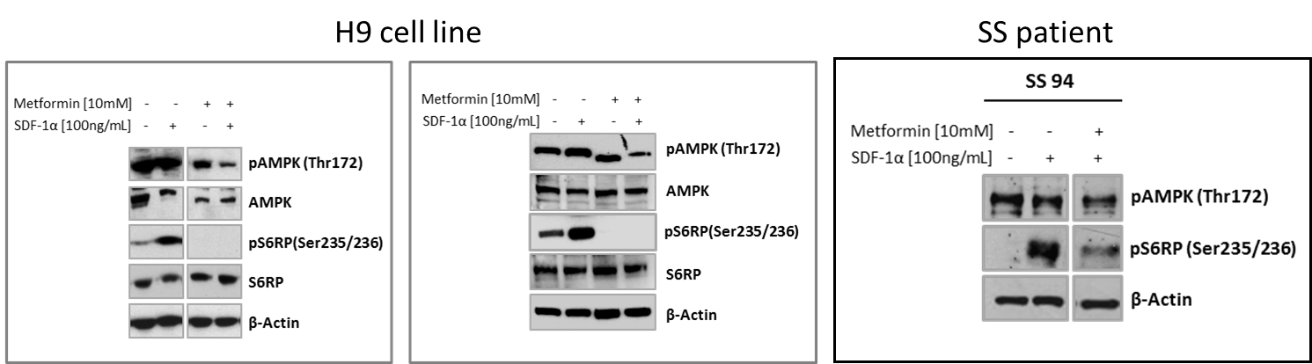
**

**Fig S7 Metformin inhibits mTORC1 activation pathway in H9 cell line and primary SS cells**. H9 cells and SS cells from SS94 patients left untreated or pretreated for 2 hours with metformin at 10 mM and then stimulated or not for 30 minutes with SDF-1 at 100 ng/ml were analyzed by WB for indicated proteins. Densitometric data normalized to β-actin were used for graph showed in Figure 6B


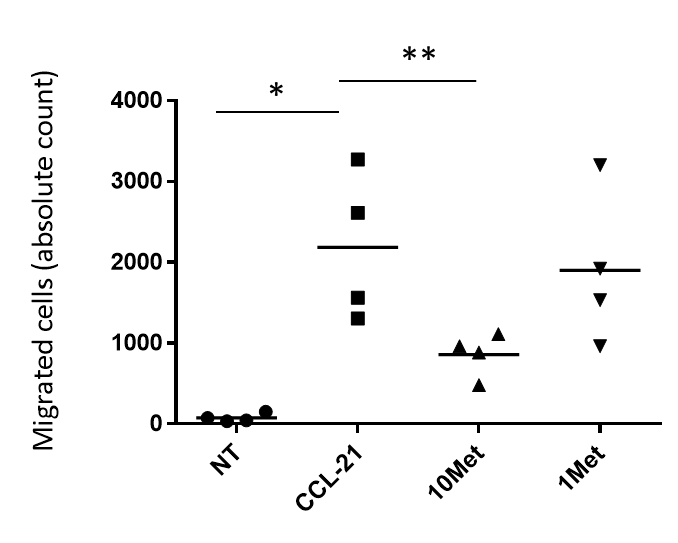


**Fig. S8 Metformin inhibits H9 cell line migrations toward CCL21.** Metformin inhibits H9 cell line migration toward CCL21. H9 (n=4) untreated or pretreated for 2 hours with metformin at 10 and 1 mM and allowed to migrate in response of CCL21 used at 300 ng/ml. Migration results are shown as absolute number of CD4+ migrated cells measured by flow cytometry. **P*= .018; ***P*= .05

**
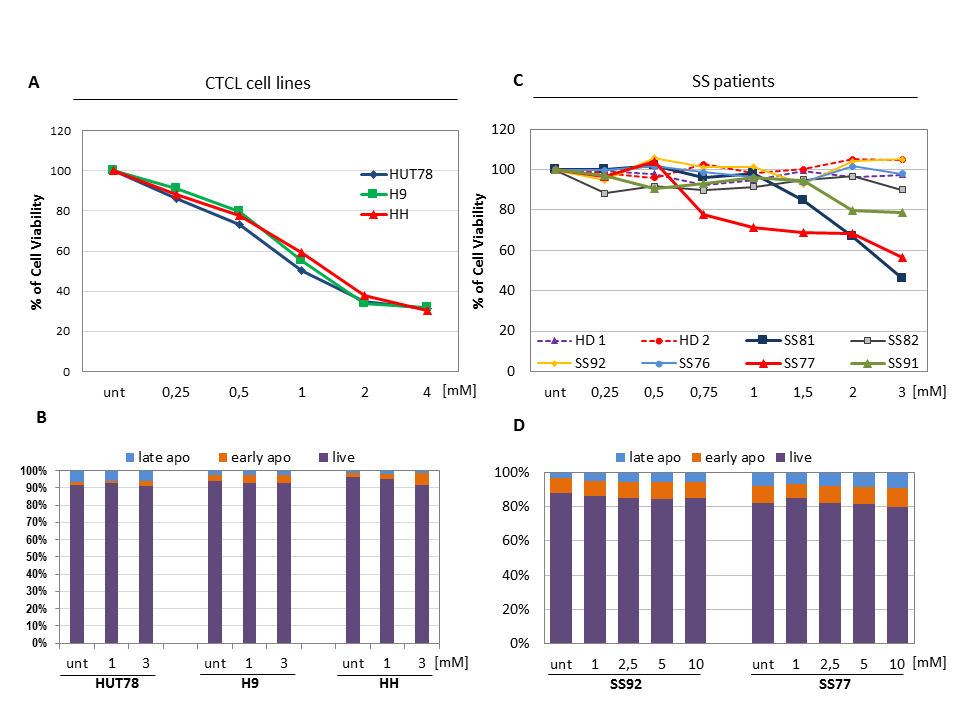
**

**Fig. S9 Effects of metformin on viability of CTCL cell lines and primary SS cells.** A) MTT assay of CTCL cell lines treated with increasing concentration of metformin for 72 hours (IC50 for Hut78=1,3 mM, for H9 and HH=1,5mM were calculated by Calcusyn program); B) CTCL cell lines exposed to metformin at the indicated concentrations for 48 hours were stained with Annexin V/propidium iodide and analyzed for apoptosis by FACS C) MTT assay of primary SS cells from 6 SS patients and 2 healthy control treated with increasing concentration of metformin for 96 hours. A moderate reduction of cells viability was only observed in two patients SS77 and SS81 showing high absolute number of large undefined (LUC) cells (Table S2) potentially referred as more activated neoplastic cells^5^; D) SS cells from two patients were exposed for 48 hours to metformin at indicated concentrations. Cells were then stained with Annexin V/propidium iodide and analyzed for apoptosis by FACS. Lymphocytes were recognized by physical parameters and gated on a FSC/CD4 plot. CD4+ were gated in an Ax/PI plot. Early apoptotic cells were identified by Ax+/PI-, late apoptotic by Ax+/PI+ and live cells by Ax-/PI- staining. Flow cytometry was performed on FACSCalibur using CellQuestPro software Percentages of AnnexinV/PI are indicated.

**SUPPLEMENTARY REFERENCES**

1 Scala E, Abeni D, Pomponi D, Narducci MG, Lombardo GA, Mari A *et al.* The role of 9-O-acetylated ganglioside D3 (CD60) and {alpha}4{beta}1 (CD49d) expression in predicting the survival of patients with Sezary syndrome. *Haematologica* 2010; **95**: 1905–12.

2 Soares A, Govender L, Hughes J, Mavakla W, de Kock M, Barnard C *et al.* Novel application of Ki67 to quantify antigen-specific in vitro lymphoproliferation. *Journal of immunological methods* 2010; **362**: 43–50.

3 Cristofoletti C, Picchio MC, Lazzeri C, Tocco V, Pagani E, Bresin A *et al.* Comprehensive analysis of PTEN status in Sezary syndrome. *Blood* 2013; **122**: 3511–20.

4 Narducci MG, Scala E, Bresin A, Caprini E, Picchio MC, Remotti D *et al.* Skin homing of Sézary cells involves SDF-1-CXCR4 signaling and down-regulation of CD26/dipeptidylpeptidase IV. *Blood* 2006; **107**: 1108–15.

5 Clark RA, Shackelton JB, Watanabe R, Calarese A, Yamanaka K -i., Campbell JJ *et al.* High-scatter T cells: a reliable biomarker for malignant T cells in cutaneous T-cell lymphoma. *Blood* 2011; **117**: 1966–1976.
